# The floral morphology of *Pseudosasa nanunica* (Poaceae, Bambusoideae)

**DOI:** 10.3897/phytokeys.271.183110

**Published:** 2026-02-11

**Authors:** Zheng-Yang Niu, Qiao-Mei Qin, Jing-Bo Ni, Zhuo-Yu Cai, Yi-Hua Tong, Nian-He Xia

**Affiliations:** 1 State Key Laboratory of Plant Diversity and Specialty Crops & Guangdong Provincial Key Laboratory of Digital Botanical Garden, South China Botanical Garden, Chinese Academy of Sciences, Guangzhou, 510650, China State Key Laboratory of Plant Diversity and Specialty Crops & Guangdong Provincial Key Laboratory of Digital Botanical Garden, South China Botanical Garden, Chinese Academy of Sciences Guangzhou China; 2 South China National Botanical Garden, Chinese Academy of Sciences, Guangzhou, 510650, China South China National Botanical Garden, Chinese Academy of Sciences Guangzhou China; 3 Guangdong Eco-engineering Polytechnic, Guangzhou 510520, China Bamboo Research Institute, Nanjing Forestry University Nanjing China; 4 Co-Innovation Center for Sustainable Forestry in Southern China, Nanjing Forestry University, 210037, Nanjing, China Co-Innovation Center for Sustainable Forestry in Southern China, Nanjing Forestry University Nanjing China; 5 Bamboo Research Institute, Nanjing Forestry University, 210037, Nanjing, China Guangdong Eco-engineering Polytechnic Guangzhou China

**Keywords:** Arundinarieae, synflorescence, taxonomy, three-branched bamboos

## Abstract

*Pseudosasa nanunica*, a bamboo endemic to Guangdong and Hunan provinces, China, has a complicated taxonomic history due to lack of information on its floral morphology. It was initially described as a member of *Indocalamus*, and later successively transferred to *Pseudosasa*, *Arundinaria* and *Acidosasa* by different researchers. With the newly collected reproductive materials, we confirmed that this species is characterized by having branch complement with 1–3 branches per node, branch base appressed to the culm, narrowly trullate culm bud, panicle-like unit of inflorescence of the synflorescence and each floret with three stamens and three stigmas, which match well with the diagnostic characters of *Pseudosasa*. After comparison with similar congeneric species, we concluded that it is a distinct species of *Pseudosasa*. A supplementary description on its floral morphology as well as two color plates and a diagram of its synflorescence is also provided.

## Introduction

*Pseudosasa nanunica* (McClure) Z.P.Wang & G.H.Ye is a bamboo native to South Hunan and Guangdong of China (Chen et al. 1996). However, due to lack of reproductive materials, the generic designation of this species has been controversial for a long time. Initially, McClure described this bamboo as a member of *Indocalamus* Nakai based on a vegetative collection, namely *Indocalamus nanunicus*McClure (1940). As noted in the protologue, McClure mentioned that the potted plant of this species is with one branch on each culm node and the mature plants with 3 branches on each culm node. Since the 1980s, all the Chinese bamboo taxonomists have agreed that this species is not a member of *Indocalamus*. Then there were two controversial opinions on the generic concepts of *Arundinaria* Michaux. Chao and Chu (1980) placed it in the genus *Arundinaria* sensu lato. In contrast, Wang and Ye (1981) put it in the genus *Pseudosasa* Makino ex Nakai because they considered that *Arundinaria* is confined to North America and does not occur in East Asia.

During study on the relationship between *Metasasa* W.T.Lin and *Acidosasa* B.M.Yang, Yang and Chao (2001) thought that *Metasasa* is very similar to the genus *Acidosasa* in inflorescences, culm leaves and foliage leaves. Thus, they agreed with Li (1997) to treat *Metasasa* as a synonym of *Acidosasa*. Yang and Chao (2001) also pointed out that the vegetative morphology of *P. nanunica* is identical to *Metasasa carinata* W.T.Lin, hence treating *P. nanunica* and *M. carinata* as conspecific. As the epithet “*nanunica*” predates “*carinata*”, they combined *P. nanunica* as *Acidosasa nanunica* (McClure) C.S.Chao & G.Y.Yang. Zhu et al. (2006a) accepted the treatment of Yang and Chao (2001). Thus, they applied the floral morphology of *M. carinata* to *P. nanunica* in their account of *Pseudosasa* in “Flora of China”. However, after field study, Zhang and Li (2010) argued that *M. carinata* and *P. nanunica* are morphologically different in the number of branches at each node (2 vs. 3), culm leaf blades (reflexed vs. erect), and length of culm leaf and foliage ligules (3–5 mm vs. 7–9 mm). Thus, they treated them as two distinct species, made a combination *Acidosasa carinata* (W.T.Lin) D.Z.Li & Y.X.Zhang, and restored the name *Pseudosasa nanunica* (McClure) Z.P.Wang & G.H.Ye based on morphological evidence. We do agree with Zhang and Li (2010) that *P. nanunica* is a species of *Pseudosasa*, as its many vegetative features, such as three subequal branches per node, branches appressed to culms, culm leaf sheaths shorter than internodes, and ligules of the culm sheaths 7–9 mm long, suggest an affinity with other congeneric *Pseudosasa* species. But it is worth noting that the floral description of this species in “Flora of China” actually belongs to *Acidosasa carinata*, and the true floral morphology of this species still remains unknown before this study.

During our fieldwork in Yingde City and Ruyuan County of Guangdong Province, we encountered two populations of flowering bamboo with leptomorph rhizomes, pluricaespitose culms that are 1–4 m high, ca. 1 cm in diameter, branch complement with 1–3 branches at each culm node, and branch base appressed to the culm, which suggest that it should belong to *Pseudosasa*. After comparison of the specimens we collected and possibly related species, we found it matches the type and description of *P. nanunica* in having abaxial surface of culm leaf sheaths with dense brown setae, erect and linear-lanceolate culm leaf blades, undeveloped culm leaf auricles and oral setae, culm leaf ligules 7–9 mm long, 2–4 foliage leaves per ultimate branch, foliage leaf ligules 7–17 mm long and relatively large foliage leaf blades (up to 30 cm long). Therefore, we concluded that this bamboo is *P. nanunica*. Notably, bamboo flowers are usually rare to see due to their long (sometimes 100–150 years) and typically monocarpic life cycle (Janzen 1974; Stapleton et al. 2009; Hodkinson et al. 2010; Ma et al. 2017). We were very lucky to obtain the flowering materials of *P. nanunica*, and a supplementary description for its floral morphology is provided here.

## Materials and methods

The descriptions were based on both living plants and dried specimens. Photos of macro-morphological organs were taken by two cameras (CANON EOS 60D & OLYMPUS TOUGH TG-6). Flowering materials (voucher: QQM-222) were dissected under a stereomicroscope (Mshot-MZ101, Guangzhou Micro-shot Technology Co., Ltd, Guangzhou, China) and images of tiny structures were taken with the attached camera on the stereomicroscope. Measurements were taken using a ruler or micrometer. Terminology refers to McClure (1940), Li et al. (2006), Beentje (2016), Cai and Xia (2021, 2024).

## Results and discussion

**Figure 1.**
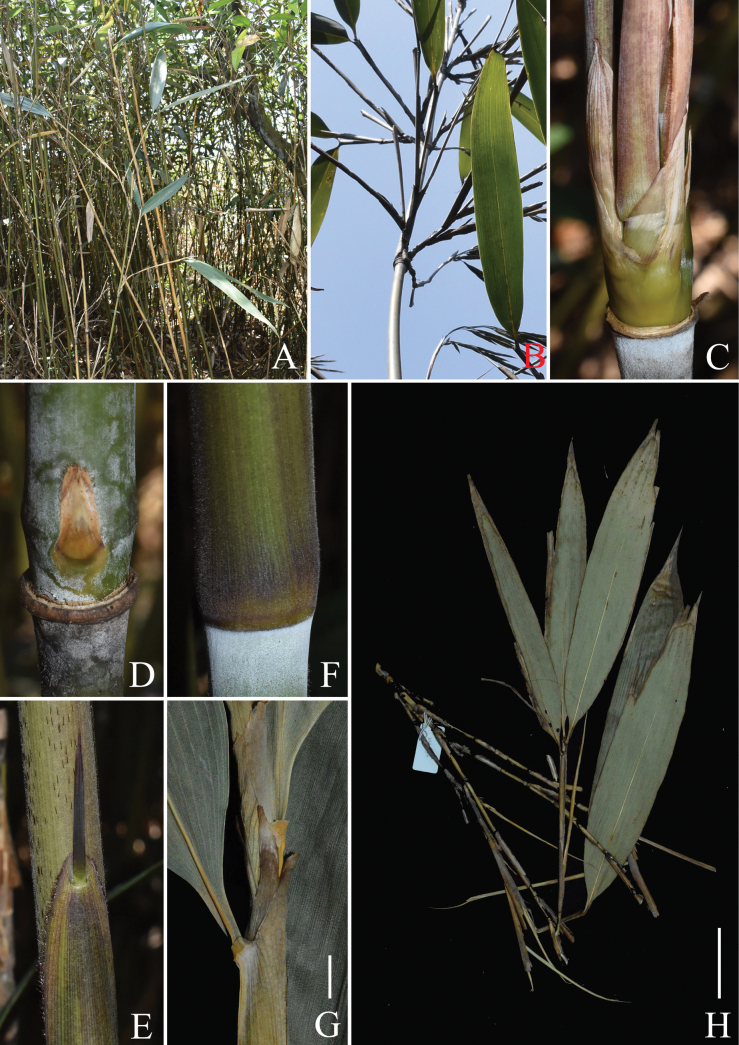
*Pseudosasa nanunica*. **A**. Habit; **B, C**. Branch complement with three branches; **B**. Showing branch base appressed to the culm; **D**. Culm bud and sheath scar with a corky collar; **E**. Part of culm leaf showing blade and ligule; **F**. Culm leaf sheath base, and white powdery ring below node; **G**. Part of foliage leafy branchlet showing inner ligule; **H**. Ultimate foliage leafy branchlet. Scale bars: 5 cm (**H**); 5 mm (**G**). Based on *N.H. Xia et al. XNH-36*.

**Figure 2.**
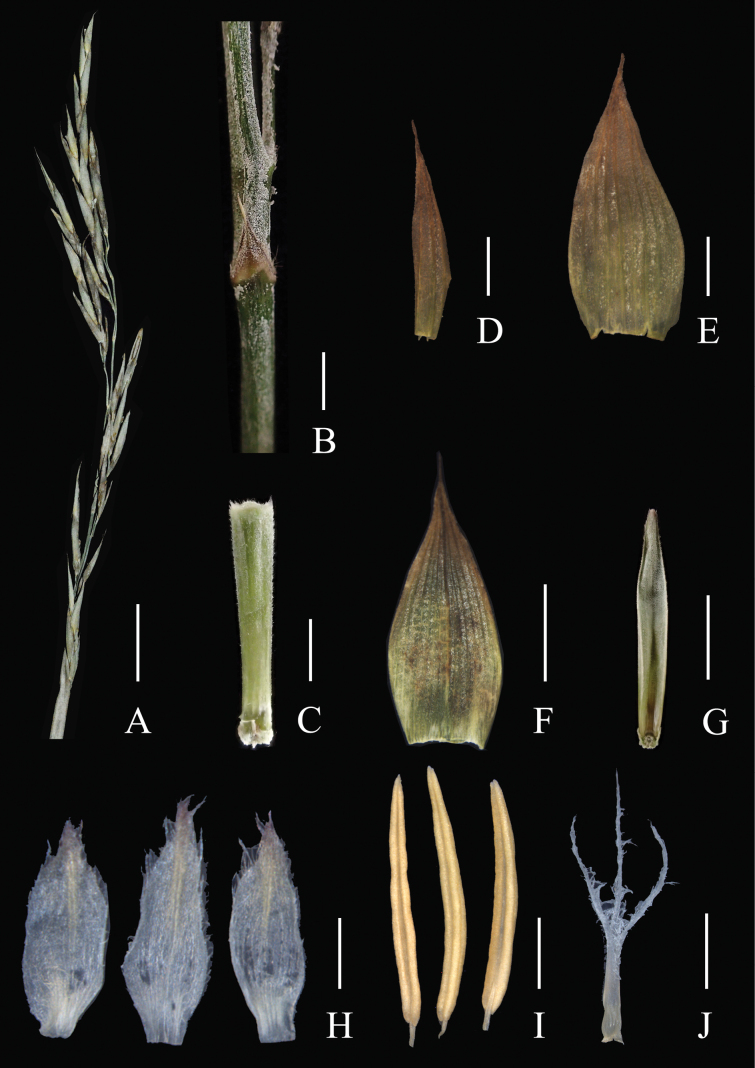
*Pseudosasa nanunica*. **A**. Unit of inflorescence of synﬂorescence; **B**. Part of main axis and a bract; **C**. Rachilla segment; **D**. First glume; **E**. Second glume; **F**. Lemma; **G**. Palea; **H**. Lodicules; **I**. Stamens; **J**. Pistil. Scale bars: 2 cm (**A**); 5 mm (**F, G**); 2 mm (**B–D, I, J**); 1 mm (**H**). **A–C, G**. Based on *N.H. Xia et al. XNH-36*; **D–F, H–J**. Based on *N.H. Xia et al. QQM-222*.

After field observation and specimen examination, we confirmed that *P. nanunica* is morphologically characterized by branch complement with solitary branch at basal culm nodes and three branches at mid or distal culm nodes, branch base appressed to the culm (Fig. 1A–C), narrowly trullate culm primary bud (Fig. 1D), panicle-like unit of inflorescence of the synflorescence (Fig. 2A), and each floret with three stamens (Fig. 2I) and three stigmas (Fig. 2J), which shows the traits of *Pseudosasa* rather than *Indocalamus*, *Arundinaria* or *Acidosasa* based on present generic concept (Nakai 1925; Chen et al. 1983, 1996; Zhu and Zhao 1996; Zhu et al. 2006a, b). Thus, the correct name for this species should be *Pseudosasa nanunica* (McClure) Z.P.Wang & G.H.Ye.

**Figure 3.**
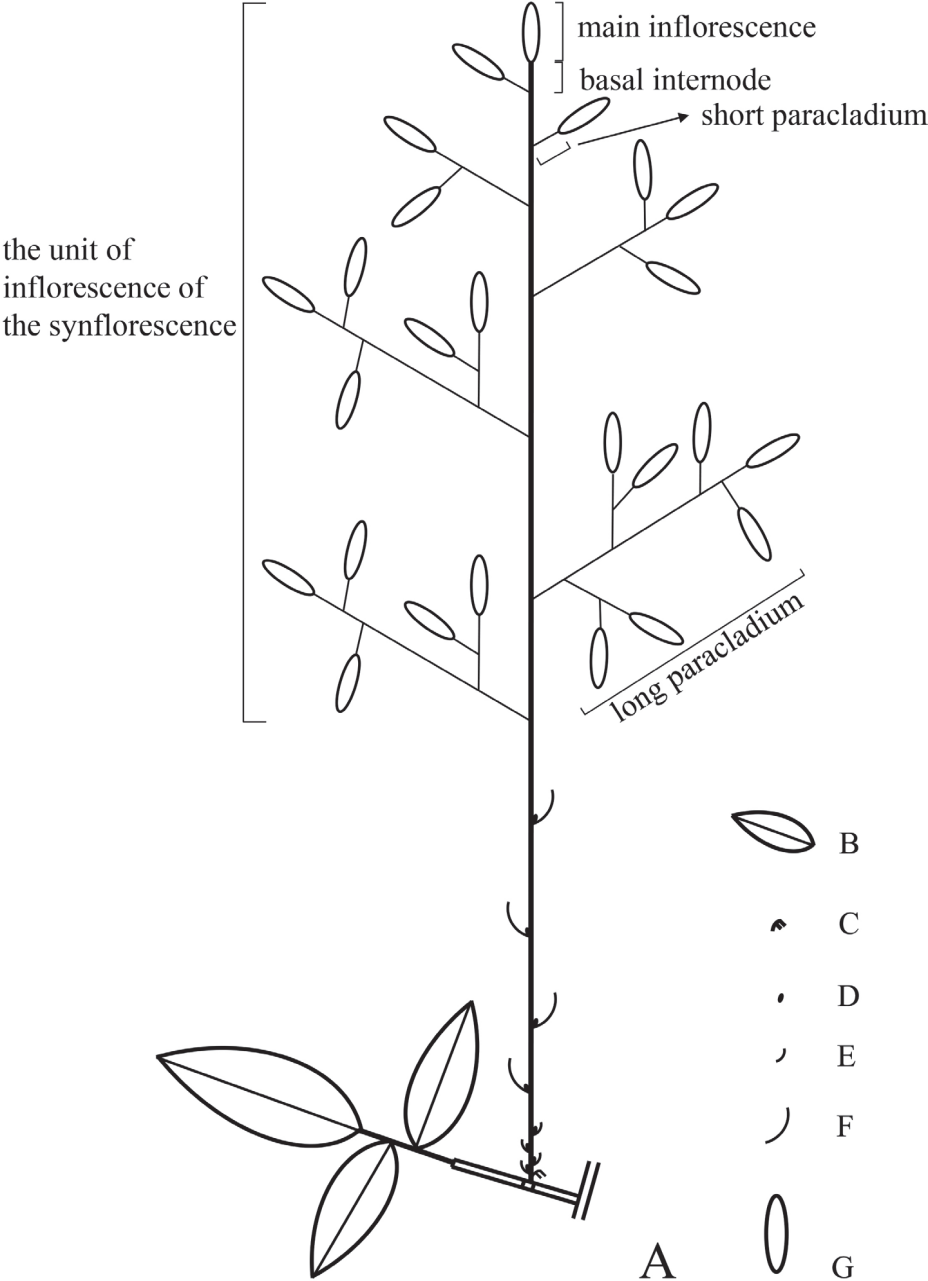
Diagram showing the synflorescence of *Pseudosasa nanunica*. **A**. A synflorescence borne at a node of leafy branch; **B**. Foliage leaf blade; **C**. Prophyll; **D**. Flowering branch bud; **E**. The branch sheath of short internode zone; **F**. The branch sheath of long internode zone; **G**. Spikelet.

According to the key of *Pseudosasa* in “Flora of China” (Zhu et al. 2006a), this species keys to couplet 9b, showing its affinity with *P. brevivaginata* G.H.Lai, *P. maculifera* J.L.Lu, *P. subsolida* S.L.Chen & G.Y.Sheng, and *P. wuyiensis* S.L.Chen & G.Y.Sheng. As the character of culm leaf sheath ornamentation mentioned in couplet 10 is not easy to observe for dried material, we re-compiled this portion of the key as below in order to help to identify *P. nanunica* and these four species after a careful morphological comparison based on protologues and descriptions from ﬂoras (McClure 1940; Lu 1981; Chen et al. 1983; Chen and Sheng 1991; Chen et al. 1996; Lai 2000; Zhu et al. 2006a). Consequently, *P. nanunica* is morphologically different from the others in having relatively long culm leaf ligules (5–9 mm; Fig. 1E) and foliage leaf ligules (7–17 mm; Fig. 1G), and relatively large size (10–30 × 2–4.5 cm) of foliage leaf blades (Fig. 1H). A newly revised description, two color plates and the synflorescence diagram of this species (Figs 1, 2, 3) are also provided here.

### Key to *Pseudosasa nanunica* and other four morphologically similar species

**Table:** 

1	Culm leaf auricles and oral setae developed; foliage leaves 6–7 per ultimate branch	***P. subsolida***
–	Culm leaf auricles undeveloped, oral setae few or undeveloped; foliage leaves 2–4 per ultimate branch	**2**
2	Foliage leaf auricles elliptic or falcate, oral setae radiate	***P. maculifera***
–	Foliage leaf auricles and oral setae undeveloped or caducous	**3**
3	Culm leaf and foliage leaf ligules short, less than 1 mm	***P. brevivaginata***
–	Culm leaf and foliage leaf ligules longer than 3 mm	**4**
4	Culm leaf ligule 3–4 mm long; foliage leaf blades 11–17 × 0.6–0.7 cm, ligule ca. 3 mm long	***P. wuyiensis***
–	Culm leaf ligule 5–9 mm long; foliage leaf blades 10–30 × 2–4.5 cm, ligule 7–17 mm long	***P. nanunica***

### Taxonomic treatment

#### Pseudosasa nanunica

Taxon classificationPlantaePoalesPoaceae

(McClure) Z.P.Wang & G.H.Ye, J.Nanjing Univ. (Nature Science) 1: 97 (1981)

0CA5C303-E373-533F-9705-3ECC3992099D

Figs 1, 2, 3

Indocalamus nanunicus McClure, Lingnan Univ. Sci. Bull. 9: 25 (1940). Basionym. ≡ Arundinaria nanunica (McClure) C.D.Chu & C.S.Chao, J. Nanjing Techn. Coll. Forest Prod. 3: 26 (1980). ≡ Acidosasa nanunica (McClure) C.S.Chao & G.Y.Yang, Acta Phytotax. Sin. 39: 66 (2001).

##### Type.

China • Guangdong: Qingyuan City, Heung Lo Keuk (Xianglujiao) Village, 13 October 1937, *F.A. McClure 20624* (holotype: US, US00065466, image!, isotype: US, US00029588, image!).

##### Description.

Shrubby bamboo. Rhizomes leptomorph. Culms pluricaespitose, erect, ca. 4 m tall and ca. 1 cm in diameter; internodes terete, initially green, yellow-green when aged, white strigose when young, glabrescent when old, initially white powdery at infranodal regions but darkened by contamination when old; walls ca. 2 mm thick; pith spongy; supra-nodal ridges raised; sheath scars prominent, corky, with persistent remains of sheath base. Intranodal regions ca. 1 cm high, thinly white powdery but darkened by contamination when old, glabrous. Culm buds solitary, narrowly trullate, yellowish green, upper parts of prophyll margins densely ciliate. Branches one at lower culm nodes, three at mid or upper culm nodes, base appressed to the culm. Culm leaf sheaths thinly leathery, shorter than internodes, tardily deciduous, initially yellowish green with purple stripes, yellow when old, abaxially densely brown setose and white pubescent, margins densely ciliate; auricles and oral setae absent; ligule arcuate, 5–9 mm tall, abaxially puberulent, margins ciliate; blades erect, lanceolate to linear-lanceolate, scabrous, apex acuminate, base slightly narrowed, margins sparsely serrulate. Foliage leaves 2–4 per ultimate branchlet; sheaths leathery, initially white powdery but darkened by contamination when old, glabrous, longitudinal ribs conspicuous when dry; auricles and oral setae absent; ligule very long, 7–17 mm tall, abaxially puberulent, sometimes white powdery, apex acute, margins sparsely ciliate; blades lanceolate, papery, 10–30 × 2–4.5 cm, apex acuminate, base cuneate, slightly oblique or not, serrulate on both sides, secondary veins 9–12 pairs, transverse veinlets conspicuous.

Synﬂorescences borne on nodes of branches. The unit of inflorescence of the synflorescence panicle-like, with 12–25 spikelets; main axis glabrous paraxially, more or less scabrous distally, densely white powdery, basal internodes 2–5 mm long; short paracladia 1–2, 3.5–7.5 mm long (excluding spikelets), slightly twisted, sparsely white pubescent, white powdery; long paracladia 3–5, 1–5.5 cm long, sparsely white pubescent, with 2–7 spikelets, lower ones usually with two second order long paracladia, sometimes with a subtending bract at base. Spikelets laterally compressed, 2–5.5 cm long; developed florets 2–6, uppermost 1–2 not fully developed; rachilla segment 6–8 mm long, with two longitudinal ridges abaxially, apex inflated, upper and middle parts densely white pubescent; glumes 2, first glume narrowly triangular to lanceolate, 4–7.5 mm long, ca. 2 mm wide near base, abaxially glabrous, 3–4-veined, apex acuminate, upper parts of margins ciliate; second glume ovate-lanceolate, ca. 10 mm long, ca. 4 mm wide near base, abaxially glabrous, 8-veined, apex acuminate, upper parts of margins ciliate, white powdery; lemma ovate-lanceolate, 1.2–2.2 cm long, 2.5–8 mm wide near base, abaxially upper parts white pubescent, other parts glabrous, white powdery, 11–14-veined, transverse veins conspicuous, apex acuminate (rarely emarginate) with an awn, awn 3–5 mm long; palea obviously shorter than lemma, 1–1.4 cm long, 2-keeled, keels and upper parts of margins white ciliate, 5-veined between keels, 4-veined outside each keel, apex acute when young, shallowly bifid when old; lodicules 3, 2–5 × 1–1.5 mm, rhomboid-lanceolate, subequal, margins densely ciliate; stamens 3; anthers yellow when mature, 6–10 mm long; ovary long ovoid, ca. 2.5 mm long, glabrous; style 1, ca 2.5 mm long; stigmas 3, plumose. Caryopsis unknown.

##### Phenology.

New shoots from April to May; flowering from April to June.

##### Distribution and habitat.

This species has been known to be endemic to South Hunan and Guangdong. It usually occurs on top of mountains and grows near streams or on shaded slopes.

##### Chinese name.

长舌茶秆竹 [cháng shé chá gǎn zhú].

##### Additional specimens examined.

**China** • **Guangdong**: Guangzhou City, Conghua District, Liuxihe National Forest Park, Wuzhishan, 11 May 2019, *N.H. Xia, Y.H. Tong & Z.Y. Cai BH-7* (IBSC); • Jiexi, Heshe, 26 June 1987, *Z.J. Feng 80595* (CANT, holotype of *Arundinaria projecta* W.T.Lin). Lechang County, 28 July 1943, *B.H. Liang et al. 84686* & *84687* (IBSC); • Lianshan County, Hedong Town, Hedong Farm, alt. 900 m, 29 April 1978, *Z.P. Wang & A.T. Liu 780036* (N); • Qingyuan City, Heung Lo Keuk [Xianglujiao] Village, 14 March 1925, *F.A. McClure 13286* (SYS, paratype of *Indocalamus nanunicus* McClure), *H. Fung 20887* (SYS, paratype of *I. nanunicus*); • Pingyuan County, 5 December 1991, *Z.J. Feng 83493* (CANT); • ibid., 10 May 1992, *Z.J. Feng 83625* (CANT, holotype of *Arundinaria bicorniculata* W.T.Lin & Z.J.Feng); • ibid., 19 May 1992, *Z.J. Feng 84098* (CANT); • Ruyuan County, 4 May 2017, *N.H. Xia, J.B. Ni, B.Q. Xu & Q.M. Qin QQM-221 & QQM-222* (IBSC); • Yingde City, Lianjiangkou, 9 Dec 1987, *M.Y. Xiao 54458* (CANT, holotype of *Acidosasa paucifolia* W.T.Lin). Shimentai National Nature Reserve, Yangmeigou, alt. 870 m, 16 April 2020, *N.H. Xia, Y.H. Tong, D.H. Cui & J.B. Ni XNH-36* (IBSC); • Zijin County, Baixi Nature Reserve, 23 May 2019, *N.H. Xia, Y.H. Tong & J.B. Ni BH-20* (IBSC). • **Hunan**: Yizhang, Mangshan, 1957, *Z.P. Wang et al. 77001* (N), *77007* (N); • ibid., 540 m elev., 24 September 1942, *B.H. Liang 83674* (IBSC); • ibid., 500 m elev., 16 October 1942, *S.Q. Chen 2516* (IBSC); ibid., 500 m elev., 20 October 1942, *S.Q. Chen 2706* (IBSC).

##### Notes.

Both Zhu et al. (2006b) and Zhang and Li (2010) cited *Acidosasa xiushanensis* T.P.Yi as a synonym of *P. nanunica*. However, according to the protologue (Yi 1992), *A. xiushanensis* is different from *P. nanunica* in having reflexed (vs. erect) culm leaf and more foliage leaves per ultimate branchlet (3–6 vs. 2–4). In addition, *A. xiushanensis* is distributed in Chongqing, which is far from Hunan and Guangdong, the center distribution area of *P. nanunica*. Thus, the identity of *A. xiushanensis* needs to be further studied.

## Supplementary Material

XML Treatment for Pseudosasa nanunica

## Additional information

### Use of AI

No use of AI was reported.

### Author ORCIDs

Zheng-Yang Niu https://orcid.org/0000-0003-0281-1504

Qiao-Mei Qin https://orcid.org/0000-0002-0567-8123

Jing-Bo Ni https://orcid.org/0000-0002-1695-6141

Zhuo-Yu Cai https://orcid.org/0000-0001-9288-0882

Yi-Hua Tong https://orcid.org/0000-0002-5034-005X

Nian-He Xia https://orcid.org/0000-0001-9852-7393

### Data availability

All of the data that support the findings of this study are available in the main text.
